# Unexpected thermal stability of two enveloped megaviruses, *Emiliania huxleyi* virus and African swine fever virus, as measured by viability PCR

**DOI:** 10.1186/s12985-023-02272-z

**Published:** 2024-01-03

**Authors:** Cecilia Balestreri, Declan C. Schroeder, Fernando Sampedro, Guillermo Marqués, Amanda Palowski, Pedro E. Urriola, Jennifer L. G. van de Ligt, Haile F. Yancy, Gerald C. Shurson

**Affiliations:** 1 Department of Veterinary Population Medicine, University of Minnesota, St. Paul, MN 55108 USA; 2 Environmental Health Sciences Division, University of Minnesota, St. Paul, MN 55455 USA; 3 Department of Neuroscience, University Imaging Centers, University of Minnesota, Minneapolis, MN 55455 USA; 4 Department of Animal Science, University of Minnesota, St. Paul, MN 55108 USA; 5 U.S. Food and Drug Administration, Center for Veterinary Medicine, Laurel, MD 20708 USA

**Keywords:** NCLDVs, Viability PCR, Thermal stability, *Emiliania huxleyi* virus, African swine fever virus

## Abstract

**Background:**

The particle structure of *Emiliania huxleyi* virus (EhV), an algal infecting member of nucleocytoplasmic large DNA viruses (NCLDVs), contains an outer lipid membrane envelope similar to that found in animal viruses such as African swine fever virus (ASFV). Despite both being enveloped NCLDVs, EhV and ASFV are known for their stability outside their host environment.

**Method:**

Here we report for the first time, the application of a viability qPCR (V-qPCR) method to describe the unprecedented and similar virion thermal stability of both EhV and ASFV. This result contradicts the cell culture-based assay method that suggests that virus “infectivity” is lost in a matter of seconds (for EhV) and minutes (for ASFV) at temperature greater than 50 °C. Confocal microscopy and analytical flow cytometry methods was used to validate the V-qPCR data for EhV.

**Results:**

We observed that both EhV and ASFV particles has unprecedented thermal tolerances. These two NCLDVs are exceptions to the rule that having an enveloped virion anatomy is a predicted weakness, as is often observed in enveloped RNA viruses (i.e., the viruses causing Porcine Reproductive and Respiratory Syndrome (PRRS), COVID-19, Ebola, or seasonal influenza). Using the V-qPCR method, we confirm that no PRRSV particles were detectable after 20 min of exposure to temperatures up to 100 °C. We also show that the EhV particles that remain after 50 °C 20 min exposure was in fact still infectious only after the three blind passages in bioassay experiments.

**Conclusions:**

This study raises the possibility that ASFV is not always eliminated or contained after applying time and temperature inactivation treatments in current decontamination or biosecurity protocols. This observation has practical implications for industries involved in animal health and food security. Finally, we propose that EhV could be used as a surrogate for ASFV under certain circumstances.

**Supplementary Information:**

The online version contains supplementary material available at 10.1186/s12985-023-02272-z.

## Background

Members of the phylum *Nucleocytoviricota*, commonly referred to as nucleocytoplasmic large DNA viruses (NCLDVs), can infect many species across diverse kingdoms. Currently, NCLDVs include members from the families *Poxviridae, Iridoviridae, Ascoviridae, Asfarviridae, Marseilleviridae, Mimiviridae*, and *Phycodnaviridae*, as well as several lineages of unclassified viruses, such as pithoviruses, pandoraviruses, molliviruses, and faustoviruses [1–3]. These viruses infect marine algae (e.g., *Laminaria digitata* [4] and *Emiliania huxleyi* [5])*,* freshwater algae (*Chlorella* sp.) [6] protozoa (e.g., amoeba) [7, 8], insects (e.g., lepidopteran larvae) [9], crustaceans (e.g., Caribbean spiny lobster and European shore crab) [10] molluscs (e.g., abalone) [11], amphibians (e.g., wood frog and gopher frog) [12], fish (e.g., finfish) [13], reptiles (e.g., lizards and snakes) [14] and mammals (e.g., humans and pigs) [15, 16].

One ecologically significant algal virus, Emiliania huxleyi virus (EhV), is a Coccolithovirus that terminates *Emiliania huxleyi* blooms and thereby controls algal community structure, gene transfer within the species, and nutrient cycling in the ocean [5, 17, 18]. Other members of *Nucleocytoviricota* have more damaging effects on their host ecology. The ascoviruses are known to cause high mortality among economically important insect pests, thereby negatively impacting insect populations [9, 19]. The NCLDV that causes smallpox (poxviruses) afflicted humankind for millennia and may have caused more human deaths than the plague in Europe [20]. Iridoviruses have become noteworthy pathogens for farmed and wild finfish, causing huge economic losses in mariculture and threatening global biodiversity [21]. The African swine fever virus (ASFV) is an Asfivirus currently undergoing a global spread among wild boar and domestic swine populations, which is having devastating effects on animal mortality, food security, and livelihoods [22].

Members of the *Nucleocytoviricota* are also known for their resilience in the environment. For example, smallpox extracted from scabs stored in envelopes was successfully cultivated every year for 13 years after the initial collection date [23]. Similarly, ASFV can survive in blood for 6 years [24]. More extraordinarily, Pithovirus sibericum was isolated from an estimated 30,000-year-old radiocarbon-dated sample when the authors surveyed the virome of Siberian permafrost [25]. Researchers have reported that coccolithoviruses can pass through zooplankton guts, prolonging EhV’s half-life [26], while a 7000-year-old record of coccolithoviruses could be amplified from sediments underlying the Black Sea [27].

In virological terms, resilience or the capability of a virus to prolong its viability is often measured by its ability to remain infectious [28]. For animal viruses, the number of infectious virus particles is frequently quantified by using the Tissue Culture Infectious Dose (TCID_50_) cell culture-based assay. The unit of measurement is the presence of a type of cell in a virus serial dilution array that shows evidence of a cytopathic effect or cell death [29]. In unicellular systems, such as those quantifying the effects of viruses on phytoplankton or bacterial bloom events, virus lysis rates (loss of cells over time) are the preferred units to determine the kill rate of a virus in natural settings [30]. Both these systems nonetheless are used to characterize the virus infection dynamic. In the fields of Food Science, Animal Science and Medicine, the TCID_50_ assay is used to test treatments or conditions that interfere with the virus infection cycle. For example, food safety microbiologists calculate delta (D) and log reduction values, with 1 log reduction equating to 90% reduction in the initial virus titre, based on cell culture-based assays. These units of measure are used to determine efficacy of treatments for inactivating pathogens in food products or fomites [31, 32]. These assays all require the host response to quantify the infectious state of the virus.

The viability PCR or V-PCR on the other hand, utilizes viability dyes, such as ethidium monoazide (EMA) or propidium monoazide (PMA), prior to nucleic acid extraction, and PCR (for DNA viruses) or RT-PCR (for RNA viruses) to evaluate the state of the virion directly [33]. If intact, the virus is considered “viable” and thus retains the potential to cause disease or be infectious. Photoreactive dyes are membrane-impermeable dyes that selectively penetrate virions that are compromised or damaged and are thereby considered dead or non-viable [34, 35]. These dyes bind to nucleic acids which subsequently inhibit DNA or RNA from amplifying during PCR or RT-PCR amplification, respectively. Recently, viability RT-PCR was used to evaluate the viability of Porcine epidemic diarrhea virus (PEDV) exposed to heat treatments, with the goal of using this method to monitor PEDV contamination in feed and feed ingredients [36]. Others have used the V-PCR assay to compare virus viabilities isolated from different systems. For example, Shirasaki et al. [37] analyzed the infectivity reduction ratio of a plant virus that was proposed to be a novel surrogate for human enteric viruses to evaluate efficacy of thermal and free-chlorine disinfection processes. Therefore, viability PCR could be a very useful method in determining whether NCLDVs remain viable when exposed to a variety of times and temperature conditions as well as chemical treatments.

Here, we used traditional cell culturing and a quantifiable version of the viability PCR (V-qPCR) assay to evaluate the effect of temperature on EhV-86 infection and viability, respectively. Temperature experiments were carried out using either a series or specific exposure times. We used ASFV and Porcine reproductive and respiratory syndrome virus (PRRSV) that infect domesticated swine as reference controls. The ASFV served as a structurally related NCLDV reference, while PRRSV served as a genetically unrelated enveloped virus with a positive single-stranded RNA genome. Additional methods including confocal microscopy, flow cytometry, and mathematical modelling were used to validate observations made by V-qPCR that could explain the remarkable resilience and longevity of many NCLDVs observed by others in nature.

## Methods

### Cell culture and virus stocks

A culture of *Emiliania huxleyi* CCMP374 (courtesy of Dr. Martinez-Martinez laboratory, Bigelow Laboratory for Ocean Sciences, Maine, USA) was grown in Alga-Gro® Seawater Medium (Carolina Biological Supplement Company, North Carolina, USA) at 15 °C with 18 h/6 h light/dark cycle (approx. 2400 lx) until the concentration of 2 × 10^5^ cells mL^−1^ was reached. Isolate EhV-86 (also courtesy of Dr. Martinez-Martinez laboratory) was added to *E. huxleyi* at a multiplicity of infection (MOI) of 1 and grown in a 15 °C incubator until lysis was observed, which was usually after 4 days [5]. The lysate was filtered through a 0.45 µm filter (Nalgene™ Rapid-Flow™ Bottle Top Filters, ThermoFisher Scientific, MA, US) to remove cell debris. The filtered lysate was divided into aliquots, titered using flow cytometry (Additional file 1: Fig. S1) and kept in the dark at 4 °C until use.

A pathogenic ASFV strain Pretoriuskop/96/4 (Pr4), isolated from *Ornithodoros porcinus porcinus* ticks, was collected from the Republic of South Africa in 1996 and was stored at -70 °C at the Plum Island Animal Disease Center. The Pr4 isolate was propagated in primary swine macrophage cultures as previously described in detail [38]. Virus DNA was isolated from stocks of ASFV Pr4 as previously described [39] and was sequenced by Nextera XT kit in the NextSeq (Illumnia, San Diego,CA) following the manufacturer’s protocol. Sequence analysis was performed using CLC Genomics Workbench software (CLCBio, Waltham, MA) and was found to be identical to the sequence with GenBank accession number: AY261363. Sequencing was performed using Illumina NextSeq550. The ASFV stock was tested to be free of Classical swine fever virus and Foot-and-mouth disease virus prior to shipment. Virulent PRRSV L1A or 1-4-4 variant was maintained in MARC-145 cells which were used for the isolation, propagation, and enumeration experiments [40]. The PRRSV sample used in the PCR assay came from a 4.68 × 10^5^ TCID_50_ mL^−1^ lysate which was stored at − 80 °C.

### Viability treatments

For all three viruses (EhV, ASFV & PRRSV), aliquots of virus filtrates were exposed to 4 °C, 60 °C and/or 80 °C, and 100 °C for 20 min. For EhV only, additional time exposures of 10, 15, 25, 40 and 60 min and temperatures of 70 °C and 90 °C were also carried out. After treatment, the filtrate was split in two aliquots and PMAxx dye (25 up to 125 µM, final concentration, Biotium Inc, CA, US) was added to one of the aliquots, and incubated in the dark at room temperature for 10 min on a rocker for optimal mixing. The mixed sample was then exposed for 30 min to the light using a PMA-Lite device (Biotium Inc, CA, US) to cross-link PMAxx Dye to the DNA or RNA. The second aliquot that was not treated with PMAxx dye was used as a control for standard quantitative PCR (S-qPCR) or reverse transcription S-qPCR (S-RT-qPCR).

### qPCR and RT-qPCR assays

*EhV & ASFV:* A QIAamp® MinElute® Virus Spin (Qiagen, CA, US) was used to extract DNA from all the aliquots. Quantitative PCR was conducted using QuantiNova SYBR Green PCR kit (Qiagen, CA, US) using the following conditions: 2 min at 95 °C followed by 40 cycles of 5 s at 95 °C and 10 s at 60 °C (reaction mix components: SYBR Green PCR Master Mix, QN ROX Reference Dye, primer pair (Table 1), molecular grade water, and 1 µL DNA template).

**Table 1:** Primers used for qPCR and RT-qPCR

Virus	Target	Primer pair	Final conc. (µM)	References
EhV	Major capsid protein gene	F: 5'-TTCGCGCTCGAGTCGATC-'3	0.7	[41]
		R: 5'-GACCTTTAGGCCAGGGAG-3'		
ASFV	p72 gene	F: 5'-AGGAGGTATCGGTGGAGGGAAC-3'	0.35	This study
		R: 5'-ATGTCCAGATACGTTGCGTCCG-3'		
PRRSV	GP7-3'UTR region	F: 5'-TAAGTTAYACTGTGGAGTTYAG-3'	0.25	This study
		R: 5'-CGCCCTAATTGAATAGGTGACTTAG-3'		

All PCR assays were conducted using a QuantStudio 3 Real-Time PCR (Applied Biosystems, Thermo Fisher Scientific, Massachusetts, USA). Standards for the EhV qPCR assays were created using EhV-86 as the template, with the major capsid protein (MCP) gene of EhV amplicon purity confirmed using an E-Gel electrophoresis system (ThermoFisher Scientific, Massachusetts, USA) and extracted using Zymoclean™ Gel DNA Recovery Kit (Zymo Research, California, USA). The number of EhV-86 genomic copies that equates to MCP copies in the extracted MCP amplicon product was calculated using the following formula:

$$No. of copies=\frac{(ng*6.022*{10}^{23})}{(83454.93 Da*1*{10}^{9})}$$

where ng is the amount of the MCP amplicon as measured by Qubit4 (Invitrogen, ThermoFisher Scientific, Massachusetts, USA), 6.022 × 10^23^ is Avogadro’s number, 83,454.93 Da is the molecular weight of our MCP amplicon as calculated using the Sequence Manipulation Suite [42] and 1 × 10^9^ is used to convert the molecular weight of the amplicon to nanograms. A dilution series of the MCP amplicon was used to create a EhV genomic equivalent standard curve as illustrated in Additional file 1: Fig. S2. Fresh dilutions for the standard curve were made for every qPCR run. Standards for the ASFV qPCR assays were created using a synthetic ASFV p72 gene designed from sequence with the accession number KM262844, positions 98,330–99,821 bp, ordered from BioGX (https://www.biogx.com/) as the template. A tenfold serial dilution of the synthetic DNA was used to create a standard curve to convert the Ct values from the qPCR assay to ASFV genomic equivalents.

*PRRSV:* RNA extraction was conducted using NucleoMag® VET (Takara Bio USA, Inc.) kit. The RT-qPCR was finally conducted using QuantiNova SYBR Green RT-PCR kit (Qiagen, CA, US) using the following conditions: 10 min at 50 °C and 10 min at 95 °C followed by 40 cycles of 15 s at 95 °C and 60 s at 60 °C (reaction mix components: QuantiNova SYBR Green RT-PCR Master Mix, QuantiNova SYBR Green RT-PCR RT enzyme, primer pair (Table 1), molecular grade water, 1 µL RNA template). Standards for the RT-qPCR assays were created by making tenfold serial dilutions of PRRSV with a known starting concentration of 4.68 × 10^5^ TCID_50_ mL^−1^ (Additional file 1: Fig. S3). All RT-qPCR assays were conducted using a Roto-gene Q Real-Time PCR (Qiagen, CA, US).

### Statistics

Statistical differences in the virus V-qPCR activity (log viable viral particles mL^−1^) between the control sample and thermally treated samples for EhV, ASFV and PRSSV were evaluated by an ANOVA test (Tukey’s test) at the 95% confidence level (*p* < 0.05).

### EhV inactivation kinetics

Experimental data on virus V-qPCR activity (log viable viral particles mL^−1^) were plotted over exposure time to obtain the virus survival curves. GinaFit software [43] was used to estimate the kinetic parameters (delta-value, expressed as the time at certain temperature for the first-log viral decline) of the Weibull models [44]. Goodness of fit of the model to the experimental data was expressed as the adjusted R^2^.

### Flow cytometry

We sampled 100 µL volumes of 0.2 µm EhV-86 filtrate exposed to temperatures of 4 °C, 60 °C, 80 °C and 100 °C for 20 min and fixed the viruses in each treatment with glutaraldehyde (0.5% final concentration). All preparations were subsequently diluted 1:100 in 1 mL final volume. Filtrates were then stained using SYBR gold (1/100,000 final dilution) and analyzed on an Accuri C6 flow cytometer (BD Biosciences, California, USA) using FL1-H threshold at 700 and fast fluidic rate (66 µL min^−1^).

### EhV-86 bioassay

A culture of *E. huxleyi* CCMP347 was grown as previously described, and once the cells reached a density of 1 × 10^6^ cells mL^−1^, the culture was divided into 900 µL aliquots, except for uninfected negative controls, and infected with 100 μL of EhV-86 previously exposed to different temperatures. Initially, each virus aliquots contained an MOI of 1 based on flow cytometry results, equal 1 × 10^4^ EhV μL^−1^. Virus treatments were conducted using 100 μL aliquots of EhV-86 at 4 °C, 37 °C, 40 °C, 45 °C, 47 °C, 48 °C, 50 °C, and 60 °C in a MiniAmp Plus Thermal Cycler (Applied Biosystems, CA, US) for intervals of seconds or minutes. Each temperature treatment at each time point had 5 replicates. Cell counts were determined on day 0, 4 and 8 day post-infection (PI) using a hemocytometer (Neubauer improved, Marienfeld, Germany) and an optical microscope (Nikon Eclipse Ci, Nikon, NY, US). Cell numbers over time were plotted as the reduction in log cell number over time of treatment (in min or sec). The lower limit for counting of *E. huxleyi* cells in a hemocytometer was 1 × 10^4^ cells mL^−1^.

### Confocal microscopy

Four samples were prepared including one non-treated control EhV-86 kept at 4 °C and three EhV-86 samples treated for 20 min at 60 °C, 80 °C, and 100 °C. Each 500 µL sample of viral lysate was incubated with DAPI dilactate (ThermoFisher Scientific, Massachusetts, USA) at a final concentration of 5 µg mL^−1^ overnight at 4 °C to stain the nucleic acids [45]. Subsequently, FM 1-43 dye (ThermoFisher Scientific, Massachusetts, USA) was added (10 µM final concentration) to the solutions which were then incubated at 4 °C in the dark for 10 min to allow the staining of the lipid membranes. The stained viral lysate was filtered through a 0.02 µm anodisc filter (Whatman® Anopore™, Sigma Aldrich, Missouri, USA) positioned on top of a disposable 0.45 µm filter apparatus previously wetted with 1 mL of dH_2_O (pump pressure 100 mbar). After filtration, the filters were dried under a flow hood in the dark, until the filters appeared opaque. Each anodisc filter was mounted in water and topped with a #1.5 cover slip. The cover slips were sealed onto the slides with clear nail polish. Images were acquired using a Nikon TiE stand equipped with an A1Rsi confocal scan head (Nikon, NY, US). DAPI was excited with a 405 nm laser, emission collected between 430 and 470 nm. FM 1-43 was excited with a 488 nm laser and emission collected between 575 and 625 nm. Channels were acquired sequentially with a Plan Apo Lambda 60 × oil, 1.4 NA objective, scan average 16, 2.2 micros/px, 90 nm pixel size and confocal aperture set at 1.2AU (28 µm). Three images were obtained for each filter. Every image was analyzed with Fiji software (https://imagej.net/Fiji), pre-processed using ‘Subtract background, 50 pixels’ to both channels and segmented with DiAna plugin after application of ‘Median’ filter for the DAPI channel and ‘None’ for the FM 1-43 channel. The final co-localization and fluorescence intensity analysis was obtained with DiAna plugin which utilized the mask of the DAPI channel to quantify the signal in the DAPI and FM 1-43 channels for each detected particle. We defined the average of the mean particle intensity derived by DiAna from the background corrected images as Relative Fluorescence Units (RFU).

## Results

### Thermostability of infectious EhV

**Fig. 1:**
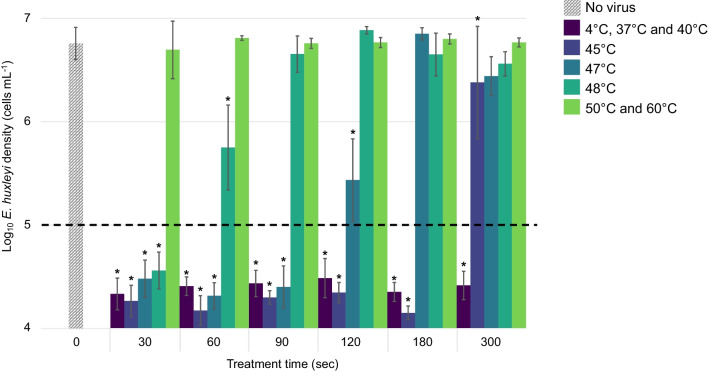
Infectivity of EhV-86 after temperature treatment. A log_10_ *E. huxleyi* cell density (cells mL^−1^) plot indicating the capacity of temperature-treated EhV to induce *E. huxleyi* lysis, with subsequent change in cell density in infected cultures eight days post-virus inoculation. EhV-86 was treated for short time-intervals: 30–300 s and at temperatures from 4 to 60 °C. Bars show the standard deviation (SD) and the mean of five replicates. Asterisks represent differences between control and treated samples by ANOVA (Tukey’s test) at 95% confidence level. The dashed line represents the lowest accurate limit of detection for cell number enumeration

To evaluate the thermal tolerance, we exposed EhV to various temperatures for various time intervals. Bioassay infectivity conditions were defined as at least a 2.5 log (corresponding to 300-fold) reduction of an *E. huxleyi* cell culture density by an EhV strain 86 (EhV-86) inoculum, four to eight days post-infection (Fig. 1). Treating EhV-86 (0.8 × 10^8^ EhV particles as measured by flow cytometry, Additional file 1: Fig. S1) with temperatures ranging from 4 to 60 °C for intervals of 0.5–5 min affirmed routine lab observations that EhV-86 remained infectious at 4 °C, 37 °C, and 40 °C (Fig. 1, Additional file 1: Fig. S4). As temperatures reached 45 °C and above, there was a decrease (*p* < 0.0001) in EhV-86 infectivity (Fig. 1). Thermal treatments at 47 °C and 48 °C resulted in reduced infectivity after 120 (*p* < 0.0001) and 60 (*p* < 0.0001) seconds, which resulted in an average reduction of host cell densities of 1.32 log and 1.01 log, respectively. Temperatures of 50°C and 60°C were sufficient to abolish viral infection after 30 s (Fig. 1).

### Viability of EhV, ASFV and PRRSV at elevated temperatures

**Fig. 2:**
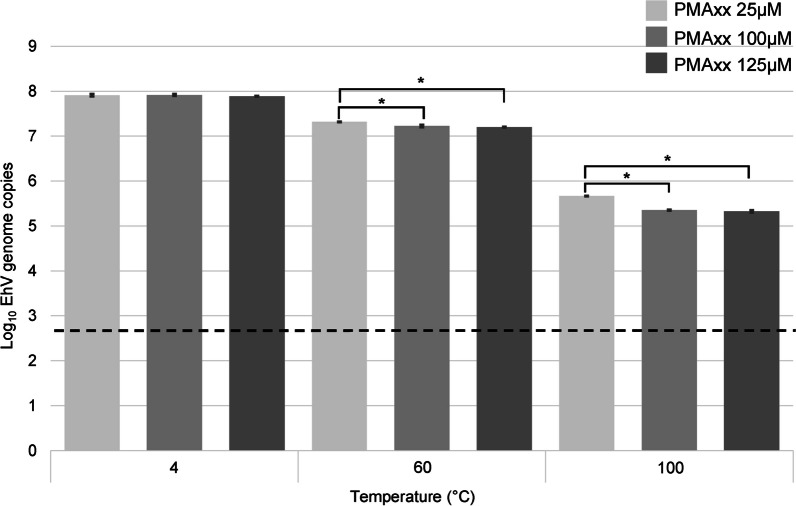
Viability qPCRs based on one hundred million initial EhV-86 particles treated at temperatures ranging from 4 to 100 °C for 20 min, using three PMAxx dye concentrations. Log_10_ mean virus counts and SD are calculated from three biological replicates. Asterisks represent differences between samples by ANOVA (Tukey’s test) at 95% confidence level. The dashed line represents the lowest limit of quantification for our qPCR assay (Additional file 1: Fig. S2)

To further characterize the effect of high temperatures on EhV-86 particles, V-qPCR was used to elucidate the infection bioassay observations. We compared untreated infectious viruses (stored at 4 °C) and non-infectious viruses (treated at 60 °C and 100 °C for 20 min), with three concentrations of the viability dye PMAxx (25 µM, 100 µM and 125 µM, Fig. 2). We observed no difference (*p* = 0.68), with less than a 0.03 log reduction in virus viability between the lowest and highest PMAxx concentrations for the 4 °C samples. While differences were observed between the 25 µM and the two higher PMAxx concentrations (at 60 °C or 100 °C), the largest difference was observed in the log reductions from 4 to 100 °C (Fig. 2). Less than one log reduction (0.60–0.72) was observed for 60°C. Moreover, a 2.57–2.59 log reduction was observed for the 100 µM and 125 µM dyes used in the 100 °C treatment.

**Fig. 3:**
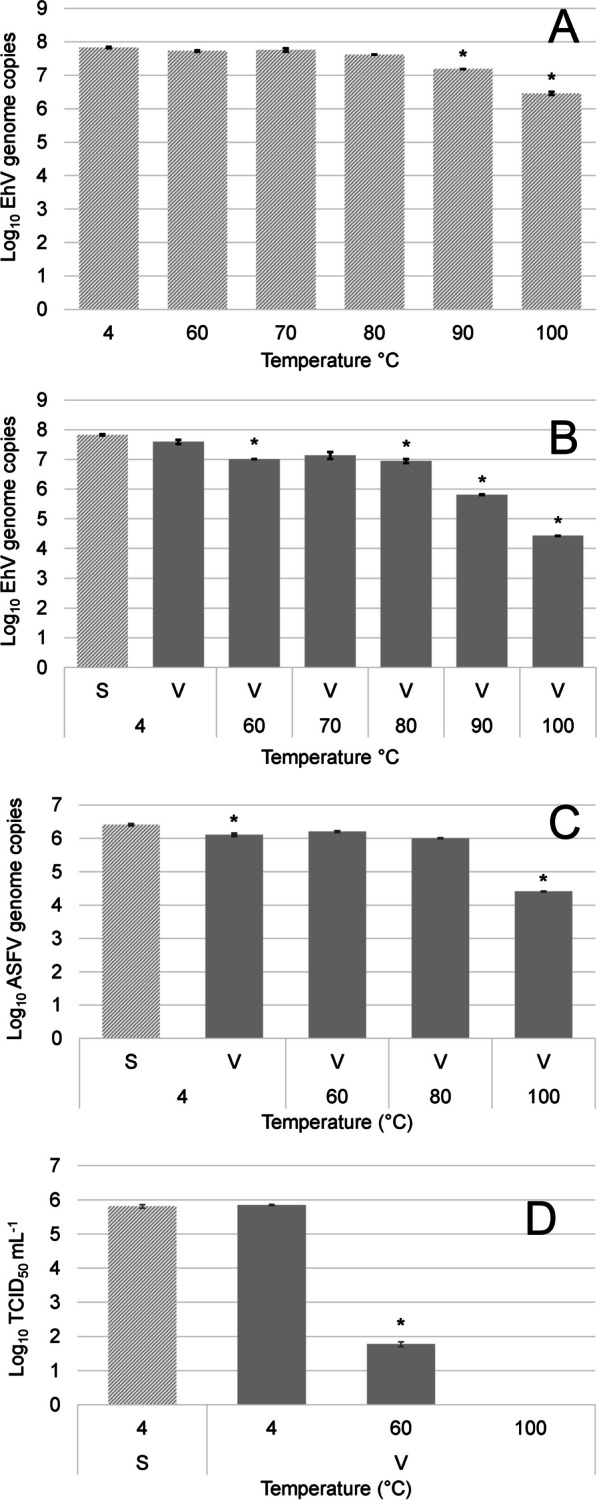
Inactivation kinetics of EhV, ASFV and PRRSV. **A** S-qPCR and **B** V-qPCR results of EhV-86 (titer 1 × 10^6^ μL^−1^) exposed to temperatures ranging from 4 to 100 °C for 20 min. **C** V-qPCR results of ASFV (titer 1 × 10^5^ μL^−1^) exposed to temperatures of 4 °C, 60 °C, 80 °C and 100 °C for 20 min. **D** V-RT-qPCR results of PRRSV (10^3^ TCID_50_ μL^−1^) exposed to temperatures of 4 °C, 60 °C and 100 °C for 20 min. In the absence of genomic standards for PRRSV, Cq data was converted to TCID_50_ units as described in the Material and Methods. Mean virus counts and SD from three biological replicates shown as measured by S-qPCR or S-RT-qPCR (light grey) and V-qPCR or V-RT-qPCR using 100 µM PMAxx final conc. (dark grey). Asterisks represent differences between control and treated samples by ANOVA (Tukey’s test) at 95% confidence level

We also wanted to determine whether the PMAxx dye had any effect on the PCR chemistry, i.e., that we could recover the anticipated genome equivalent amounts based on the amount of virus particles tested. We found that the S-qPCR assay produced the expected virus genome copies for all the viruses when stored at 4 °C (Fig. 3). We observed that even when EhV-86 was exposed to temperatures up to 80 °C for up to 20 min, no difference was found compared to the control (*p* > 0.05) and less than a 0.25 log reduction in the number of total virus genomes occurred (Fig. 3A). Temperatures greater than 90 °C resulted in a significant reduction (*p* < 0.0001) of the number of genomes by an additional 0.43 log, with exposure to 100 °C resulting in a 1.37 log reduction of genomes (*p* < 0.0001, Fig. 3A). The use of V-qPCR, however, revealed a 0.88 log reduction (*p* < 0.0001) in the number of viable virus particles after exposure to 80°C (Fig. 3B). The 90 °C treatment resulted in a 2.02 log reduction (*p* < 0.0001) of the number of genomes, and a 3.40 log reduction at 100 °C (*p* < 0.0001, Fig. 3B).

Comparing the heat inactivation data obtained for EhV (Fig. 3B) to that of ASFV (Fig. 3C), as revealed by V-qPCR, showed a similar limited log reduction (no differences with the control sample, *p* > 0.05) in ASFV viable particles when exposed to 60 °C and 80 °C for 20 min. Similarly, only at 100 °C was a greater than 2 log reduction (*p* < 0.0001) in viable particles for both viruses observed. This thermal stability was not observed for PRRSV when treated at temperatures ranging from 60 to 100 °C for 20 min (Fig. 3D). At 60 °C and 100 °C treatments, greater than 4 log (*p* < 0.0001) and 5 log reductions (*p* < 0.0001), respectively, were recorded (Fig. 3D).

### Modelling thermal inactivation kinetics

**Fig. 4:**
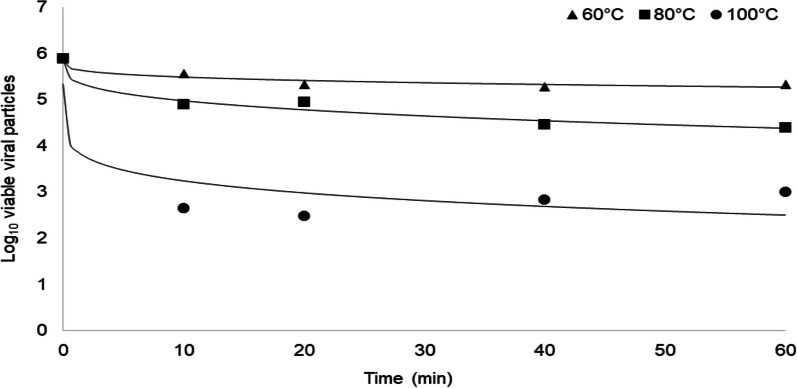
Inactivation kinetics of EhV. Temperature and time inactivation kinetics as determined from viability PCR for EhV-86 treated at 60 °C, 80 °C, and 100 °C for 10 min, 20 min, 40 min, and 60 min. A Weibull model was fitted to the data with R^2^ values of 0.93, 0.97 and 0.95, for the 60 °C, 80 °C, and 100 °C data sets, respectively

The Weibull model is a continuous probability distribution model applied over exposure time at different temperatures and was applied to report our V-qPCR for EhV inactivation results (Fig. 4) because of the nonlinear or tailing phenomena observed in the EhV data (Fig. 3A, B). The delta values (thermal resistance or time at certain temperatures to reduce 1 log of the initial virus concentration) for EhV-86 were 410.5, 14.5 and 0.02 min at 60 °C, 80 °C and 100 °C, respectively. The time required to reduce EhV-86 infectivity by 3 log was 541.7 h, 11.6 h, and 0.4 h at 60 °C, 80 °C, and 100 °C, respectively. In an effort to compare and contrast rates of EhV and ASFV inactivation, we applied the Weibull model to estimate the delta values from previously reported ASFV inactivation data based on the HAD_50_ method [46]. The delta value for ASFV was calculated to be 4 min at 60 °C, with a 3 log reduction of ASFV requiring exposure to 60 °C for 11.4 min.

### Viability as determined by confocal microscopy and flow cytometry

**Fig. 5:**
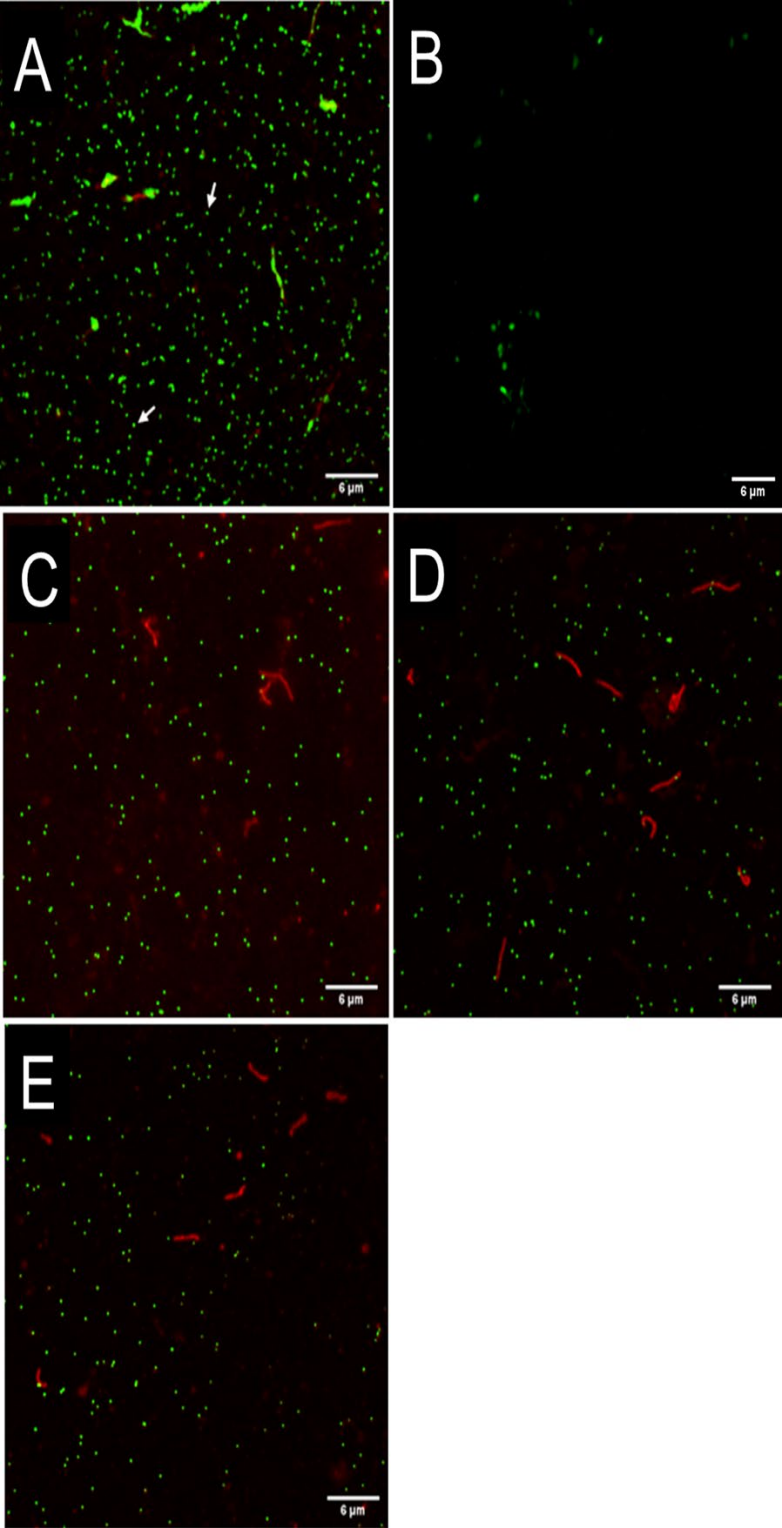
EhV-86 particles observed using confocal microscopy after temperature exposure. Each 200 nm dot (green) corresponds to one virus particle. All images are merged composites of DNA stain (DAPI, shown in green for ease of visualization) and lipid stain (FM 1-43, shown in red). The images show **A** the DNA-containing viral particles in the positive non-treated EhV (4 °C), **B** EhV-free growing medium, **C** the DNA-containing viral particles after 20 min treatment at 60 °C, **D** 80 °C and **E** 100 °C. White arrows in image (**a**) indicate an example of a virus particle

Confocal microscopy was used to confirm the structural integrity of the EhV particles after exposure to various temperatures and times because virus DNA can only be visualised if it is constrained by the virus membrane and/or capsid (Fig. 5 and Additional file 1: Fig. S5). We observed an average of 515 (SD = 44) particles per image (n = 3) for EhV-86 exposed to 4 °C, which corresponds to a virus concentration of 2.6 × 10^8^ EhV mL^−1^ (Fig. 5A), which was a concentration similar to the value obtained by flow cytometry (Additional file 1: Fig. S1). After 20 min of exposure to temperatures of 60 °C, 80 °C, and 100 °C, the average number of DNA-containing particles recorded per image was 191 (SD = 20), 195 (SD = 44) and 180 (SD = 44), respectively, corresponding to a 0.43 log reduction in the number of viral particles relative to the 4 °C treatment (Fig. 5C–E).

The mean ratio was 0.54 for fluorescence signals of DNA (stained by DAPI) and the lipid membrane (stained by FM 1-43) of EhV-86 particles at 4 °C. This ratio increased with increasing temperatures to 1.19 at 60 °C and peaked at 3.54 at 80 °C. The ratio was reduced to 1.58 when EhV-86 particles were exposed to 100 °C (Additional file 1: Fig. S5). Like the DNA-to-lipid membrane signal ratio, the average DNA signal per virus particle (DAPI) also increased with increasing temperature from 153 Relative Fluorescence Units (RFU) at 4 °C, to 351 RFU at 60 °C, and 433 RFU at 80 °C. The DNA signal was reduced to 242 RFU at 100 °C but remained greater than that observed at 4 °C (Additional file 1: Fig. S5, green line). The lipid membrane signal (FM 1-43) was similar at both 4 °C and 60 °C, measuring at 301 and 306 RFU, respectively. The lipid signal decreased at 80 °C and 100 °C to 128 and 161 RFU, respectively (Additional file 1: Fig. S5, red line).

**Fig. 6:**
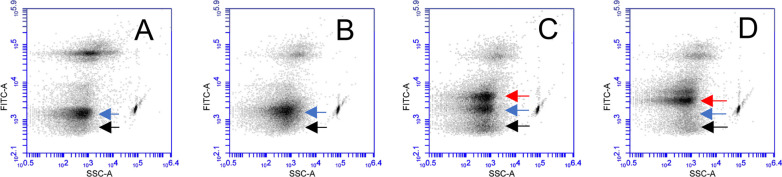
Changes in EhV-86 particle properties at elevated temperatures using flow cytometry (FC). **A–D** Fluorescein isothiocyanate (FITC)-channel vs SSC-channel plots of EhV particles treated at 4 °C, 60 °C, 80 °C, and 100 °C for 20 min, respectively. Location of infectious (black and blue) and damaged EhV-86 (red) are indicated by arrows. Spherotech beads (1.32 um) were run as a size reference in all experiments (right hand side of arrows)

In addition, flow cytometry revealed that the SYBR nucleic acid staining efficiency of EhV-86 increased with increasing temperatures (Fig. 6) as previously observed with DAPI staining (Additional file 1: Fig. S5). No discernible changes were observed in the particle properties as measured by side-scatter (SSC), which determines the light scatter at a ninety-degree angle relative to the laser (Fig. 6). Even at 4 °C, the population of EhV-86 particles was not homogeneous (Fig. 6A). When comparing flow cytometry plots of 4 °C and 60 °C, a discernible increase from 13,973 to 16,183 events per 30 µL, which corresponds to 0.47 × 10^8^ to 0.54 × 10^8^ EhV mL^−1^, respectively, in the EhV-86 population was observed (Fig. 6A, B, blue arrow). In addition, a new cluster of particles with a similar SSC appeared when viruses were exposed to 80 °C, which we characterized as damaged virions that still have DNA that can more efficiently be stained with SYBR (Fig. 6C, red arrow). These damaged particles were also observed at 100 °C (Fig. 6D, red arrow) but with reduced levels of the populations observed at 4 °C (Fig. 6, blue and black arrows). In general, the extent of the particle degradation increased with increasing temperatures.

**Fig. 7:**
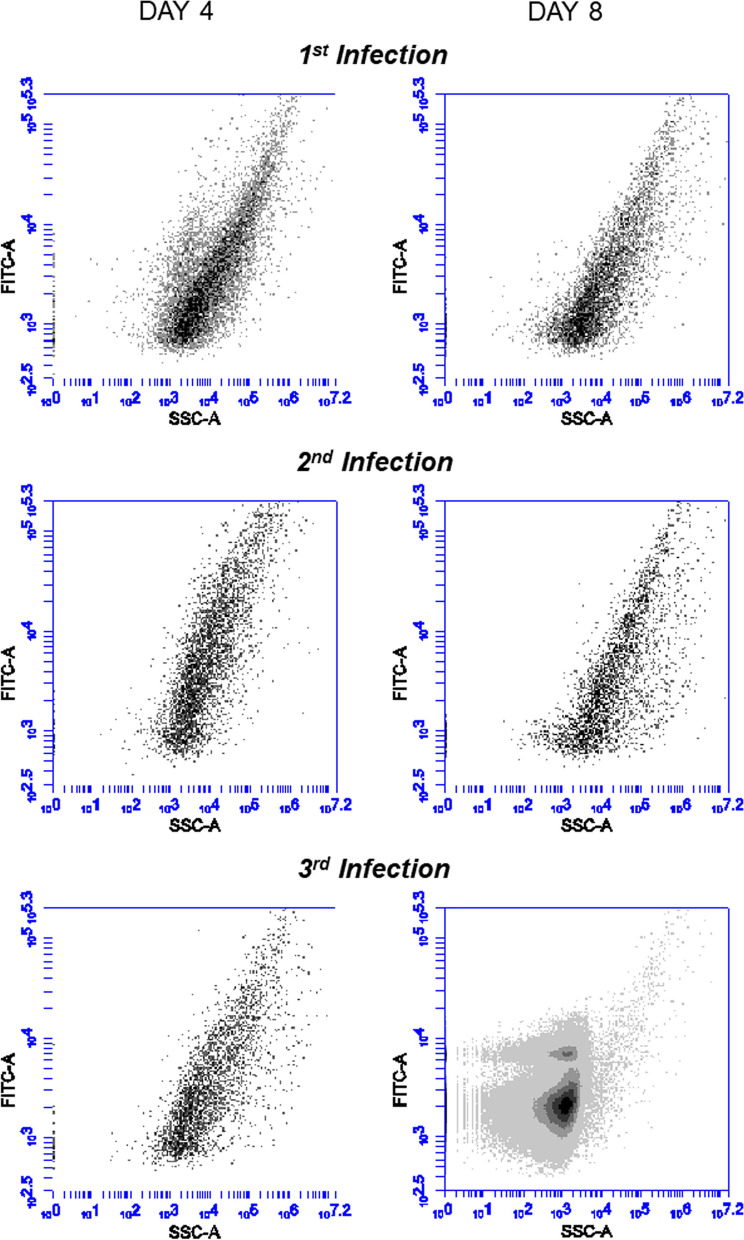
Recovery of the infectious state of EhV-86 through multiple bind passages of 50 °C in *E. huxleyi* as visualized using flow cytometry. Flow cytometry plots of 0.2 μm filtrates of three passages (rows) of *E. huxleyi* cultures after 4 and 8 days (columns) after sequentially being infected with 50 °C treated EhV-86 (Fig. 1) and day 8 filtrates from previous inoculated culture. We obtained the distinctive EhV-86 population on day 8 in the 3rd passage

Finally, we added EhV-86 treated at 50 °C for 20 min (as described in Fig. 1) to an *E. huxleyi* culture and continuously passaged it through successive bioassays. As previously observed, no lysis was observed on day 8 post-infection. Flow cytometry confirmed the absence of a dominant EhV population (neither infectious or damaged virions) on both day 4 and day 8 (Fig. 7). The 0.2 μm filtrate obtained on day 8 was used to inoculate two successive passages of *E. huxleyi* bioassays. On day 8 after the 3rd passage, the culture died, and we observed EhV-86 in the resulting external media (Fig. 7). Our untreated EhV positive controls were positive after the first passage, while our mock infected negative cultures remained virus-free after three passages.

## Discussion

Given that growth of *E. huxleyi* is optimal below 25 °C, with an upper limit of 30 °C, it is interesting to note that EhV can remain infectious 10 °C above this upper limit. In addition, even under the undesirable and devastating future climate change predictions of a sea surface temperature increase of up to 5.8 °C above current levels [47, 48], increased global temperatures will not negatively affect the ability of EhV-86, as a cell-free particle in the environment, to re-infect its bloom-forming host. This is an important observation as lytic viruses that infect microbes spend proportionally more time in the environment than inside their infected host. In addition, the thermal stability of the EhV particle itself, i.e., EhV-86 particles retain their genomes even after exposure to temperatures greater than 60 °C, was an unexpected result. This was because of the abolishment of infectivity as reported for temperatures greater than 45 °C in the bioassay experiment. These findings indicate that the virus capsid core remains intact but not infectious at temperatures greater than 60 °C when treated for 20 min. Given that only minor differences in the log reduction values between identical treatments as observed by V-qPCR were across a broad range of PMAxx concentrations tested, we can conclude that the effect of temperature on EhV viability was not a result of an assay artifact or lack of assay sensitivity. In addition, presence of genome-containing particles at 60 °C, 80 °C, and 100 °C after 20 min treatments was confirmed by confocal microscopy and flow cytometry.

The confocal microscopy data also provided new mechanistic insights into the effects elevated temperatures have on EhV virion structure. We observed that EhV-86 exposure to 80 °C resulted in most of the virions being damaged by loss of the outer membrane. Given that DAPI passes through viable cell membranes less efficiently compared with damaged cells [49], the loss of the outer membrane may have resulted in the DAPI stain penetrating the virion more efficiently, resulting in the higher DNA signal at all temperatures compared with the signal at 4 °C. Most of the damaged EhV particles after exposure to 80 °C had either a denatured capsid plus inner membrane or only the internal membrane to surround their genomes. These results suggest that the loss of the infectious state of the virus population (even at temperatures greater than 45 °C) is not a function of the loss of virus genomes from the particles, but rather the damage to the outer membrane and the associated envelope complex. We speculate that increasing the temperature to 80°C or 100 °C resulted in the loss of this outer membrane (i.e., loss in FM 1-43 signal). Most of the damaged particles had either a denatured capsid plus inner membrane or only the internal membrane to surround their genomes. This is supported by the increased DNA labelling efficiency observed at 80 °C. A loss in the DNA signal relative to 60 °C and 80 °C treatments were only observed when EhV-86 was exposed to 100 °C, indicating the likely beginning of virus DNA degradation.

The flow cytometry data also revealed a structural change to the EhV particle that also resulted in a change in permeability to the DNA binding stain. Taken together, there is a possibility that the particles with a disrupted outer membrane but with an intact capsid, inner membrane and genome could potentially infect *E. huxleyi* if acquired by phagocytosis or similar endolytic event [45, 50]. Based on the observed stability of genomes inside the damaged particles of EhV-86 and their potential role in infection, we propose that EhV is still viable after temperature exposure up to 100 °C for 20 min. The stability of NCLDV virion structures at 100 °C was previously observed for mimiviruses, specifically Samba virus, as visualized by cryoelectron microscopy [51]. Here the authors found that the virions were partially opened in only a third of the virions when treated for 1 h at 100 °C. Moreover, we observed that either a small number of infectious EhV particles survived 50°C treatment for 20 min (below the limits of detection of a single bioassay infection cycle) or that 50 °C treated EhV can trigger infection after being seemingly thermally inactivated. Future work will need to further investigate the limits of thermal recovery for EhV exposed to a wider range of temperatures and times. If this observation holds true and if we use the temperature stability data as proxy of general virion stability, it would explain why NCLDVs have been recovered after thousands of years from permafrost [52, 53] and in sediments [27].

With regards to ASFV, Plowright and Parker (1967) published one of the first studies to show that storing ASFV at 4 °C preserves infectivity, as determined by a cell culture-based method, of viraemic blood for at least 525 days. At 37 °C, culture medium containing ASFV remained infectious for 11–22 days, but at 60 °C it was only infectious for 30 min [46]. This observation was consistent with Montgomery’s 1921 report in which he found that ASFV was extremely resistant to high temperatures, putrefaction and desiccation [54]. As more recently reviewed by Fischer et al. [55] indicated that many cell culture-based infection studies found that ASFV infectivity, in preserved or clotted pig blood, at 4 °C remained infective for as long as 548 days. Similarly, in pig meat stored at 4–8 °C, infectious virus could be detected for up to 155 days [56]. Infected spleen samples stored in a refrigerator remained infectious for 204 days, but when buried in soil, it remained so for 280 days. Bone marrow (in boned meat) remained infectious for 180–188 days, skin and fat for 300 days, and offal for 105 days [56]. Fischer et al. (2020) also reported that when using standard PCR-based methods, ASFV genomic DNA was found in organs such as spleen, kidney and lung for up to 714 days when stored at − 20 °C, 136 days at 4 °C and 17 days at 23 °C [57]. Furthermore, ASFV genomic DNA was even found to be stable in faeces with half-lives ranging from more than 730 days at temperatures up to 12 °C, and for about 15 days at temperature of 30 °C [58]. Finally, other studies that also used the cell culture-based ASFV methods confirmed Plowright and Parker’s (1967) observations that ASFV can be thermally inactivated at temperatures greater than 50 °C [59, 60]. However, our V-qPCR provides a new understanding that the cell culture-based and standard PCR detection datasets do not provide [61]. The unexpected, enhanced stability of the ASFV particle and DNA genome at 100 °C for 20 min can only be explained by the virus DNA core being protected as a result of the multi-layered protein capsid and envelope structure that surrounds it [62]. As previously described, these protective layers could also explain ASFV longevity as observed in blood, tissue, organs and even faeces.

The Weibull model estimates indicate that previous assumptions about the inactivation kinetics of ASFV need be re-evaluated, especially those based on HAD_50_ determinations. Moreover, the endocytic pathway of macropinocytosis is constitutively active in macrophages and is a key route of entry for ASFV into host cells [63], as well as for other animal-like infecting NCLDVs [64]. Any risk assessment using inactivation kinetics data on NCLDVs should not only consider the infectious state as revealed by traditional infection experiments or bioassays but should also consider the viable status of the NCLDV studied.

Other more common swine RNA viruses, such as Porcine epidemic diarrhea virus (PEDV) and PRRSV, are comparatively less resilient to time and temperature exposure than ASFV and EhV [65, 66]. The results of the V-RT-qPCR for PRRSV obtained in our study were similar to that observed by others based on infectivity cell cultured-based data [66]. These results are not surprising because PRRSV is reliant on surface-bound membrane glycoproteins, such as GP5-M and GP2-4, for infection [67]. Therefore, as is typically observed for enveloped viruses, neutralizing these surface virus proteins will render RNA viruses non-infectious [68]. In addition, the largely intact and potentially viable particles are unable to infect host cells because infection occurs solely through a receptor-mediated route.

Atypical routes of infection can occur as the genomes of some viruses, if acquired by injection, are able to infect and create new, fully functional virus progeny [69]. Such atypical infection routes of contagious DNA were previously confirmed for ASFV [70]. Although the hemadsorption (HAD_50_) cell culture-based assay is commonly used to determine virus concentrations and evaluate ASFV infectivity [55]. This assay is reliant on the virus encoded CD2-like glycoprotein surface protein being present in the outer membrane of an ASFV-infected macrophage because it mediates hemadsorption [71]. This outer cell-derived membrane also becomes the outer envelope of the ASFV particle, giving the particle itself red blood cell or hemadsorption properties [72]. However, as shown for both EhV and ASFV, temperatures greater than 60 °C result in the virus particle remaining intact or viable even if the surface glycoprotein becomes denatured, which causes an inaccurate assessment using conventional cell culture-based assays. In the case of ASFV, these heat damaged ASFV particles may still be “infectious” because intact surface glycoproteins are not required for accidental phagocytosis [71]. In addition, Andrés [73] proposed that non-receptor mediated micropinocytosis could be a key process enabling ASFV entry into host cells. Even infectious cell culture-based methods (e.g., TCID_50_ or HAD_50_) may be incapable of quantifying the true infectivity of ASFV, especially if Vero cells and not porcine-derived macrophages are used in these assays [74]. Moreover, ASFV thermal stability was recently confirmed [75]. Here the authors developed a vaccine for ASFV by heat treating ASFV to 60 °C for 2h. Despite the cell culture results suggesting complete inactivation, animal bioassays resulted in viremia and sera conversion. Therefore, ASFV was still infectious after the 60 °C for 2 h treatment, although is their serendipitous case ASFV was attenuated. What is clear is that cell culture only data can produce contradictory and misleading data. Consequently, the use of a combination of multiple assays that also includes the V-PCR assay overcomes these shortcomings, especially if the virus particle is entirely destroyed, as it does not rely on the host cell to determine whether the virus has the potential to infect.

## Conclusion

The thermal stability, based on V-qPCR data, for both NCLDVs (included in this study) provides unprecedented new insight into the true resilience of NCLDVs in the environment. Furthermore, with the ongoing outbreaks of ASF across Europe and Asia, and more recently in the Dominican Republic, we must now consider a third scenario that ASFV persists stably in an altered but viable state in an environment other than its host, as well as in previously known reservoirs or alternate hosts provided by soft ticks and wild pigs [76]. Current mitigation or inactivation methods that rely solely on cell culture-based methods data to confirm ASFV inactivation will not account for the true viability and thus long-term infection potential of ASFV. Could this be why ASFV is not always eliminated or contained after applying time and temperature inactivation treatments in current decontamination or biosecurity protocols? Confirming a viable state for ASFV could have unprecedented implications for businesses directly or indirectly connected to the swine industry, including the global feed industry [61].

To our knowledge, here we show for the first time, how an enveloped virion anatomy is not an automatic weakness as often observed in other enveloped RNA virus systems (i.e., the viruses causing PRRS, COVID-19, Ebola, or seasonal influenza) [77]. In addition, EhV could be used as a safe surrogate for ASFV to evaluate inactivation kinetics on fomites or in various feed ingredient matrices when subjected to thermal and chemical mitigation treatments. Given its similar stability profile to that of ASFV and that it also does not require a receptor-mediated mechanism for infection, EhV could serve as a surrogate in future viability-based experiments. We successfully used EhV in a transport study that confirmed previous modelling and lab-based data that ASFV can retain viability in swine feed matrices during long-term transport across the continental United States [78].

### Supplementary Information

**Additional file 1: Fig. S1.** Quantifying EhV-86 using flow cytometry; **Fig. S2.** Example of a standard curve used in qPCR assays; **Fig. S3.** The standard curve created using a PRRSV TCID_50_ mL^−1^ tenfold dilution series and the Cq values obtained from RT-PCR; **Fig. S4.** Infectivity of EhV-86 after temperature treatment; **Fig. S5.** Efficiency of staining EhV-86 at different temperatures using a nucleic acid (DAPI) and lipid (FM 143) stain.

## Data Availability

Not applicable.

## Abbreviations

NCLDVs: Nucleocytoplasmic large DNA viruses
EhV: *Emiliania huxleyi* Virus
ASFV: African swine fever virus
PRRSV: Porcine reproductive and respiratory virus
V-qPCR: Viability quantitative PCR
S-qPCR: Standard quantitative PCR
PMA: Propidium monoazide
