# Supplementary material for: Unexpected thermal stability of two enveloped megaviruses, Emiliania huxleyi virus and African swine fever virus, as measured by viability PCR
Source: Virol J. 2024 Jan 3;21:1. doi: 10.1186/s12985-023-02272-z (PMC10765680; doi:10.1186/s12985-023-02272-z)
Supplement: Supplementary file 1 — Additional file 1: Fig. S1. Quantifying EhV-86 using flow cytometry; Fig. S2. Example of a standard curve used in qPCR assays; Fig. S3. The standard curve created using a PRRSV TCID50 mL−1 tenfold dilution series and the Cq values obtained from RT-PCR; Fig. S4. Infectivity of EhV-86 after temperature treatment; Fig. S5. Efficiency of staining EhV-86 at different temperatures using a nucleic acid (DAPI) and lipid (FM 143) stain. [file 12985_2023_2272_MOESM1_ESM.pdf]

-

Supplementary Information

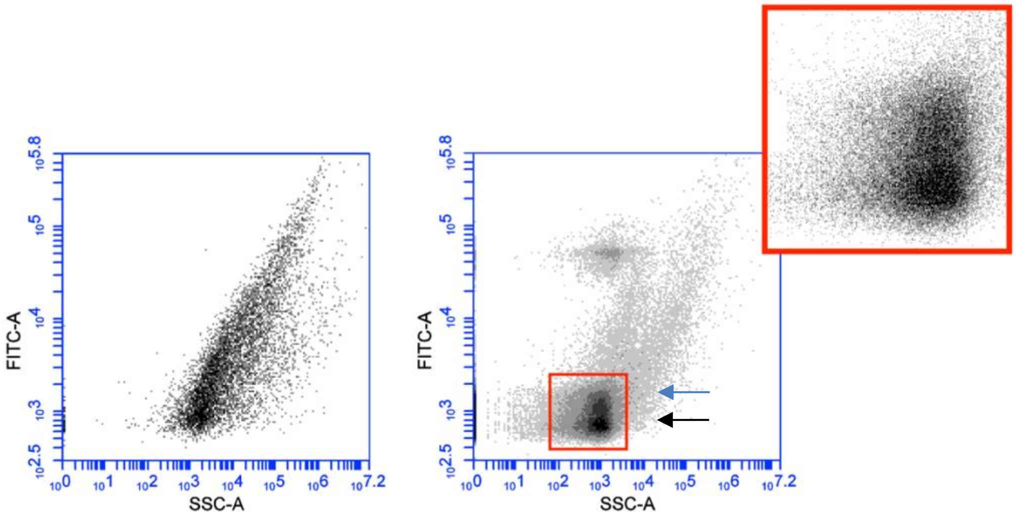

Fig S1. Quantifying EhV-86 using flow cytometry. Flow cytometry plots of 0.2 μm filtrate of (A) an uninfected *Emiliania huxleyi* culture and (B) *E. huxleyi* dead culture 4 days after being infected with EhV-86. EhV-86 was maintained at 4°C before adding to *E. huxleyi* culture. Filtrates were fixed with 0.5% glutaraldehyde and subsequently diluted 1:100. Filtrates were then stained using SYBR gold as described in [1]. Flow cytometry enumeration based on green fluorescence (FITC-A) and side scatter (SSC-A) allowed us to identify and gate EhV particles (red box) as previously described by [2]. We obtained a virus count of 37,335 in 50 µL, which after taking into account the previous 100x dilution, correspond to 0.8 x 10^8 EhV mL^{-1}. Two regions of high (blue arrow) and low (black arrow) SYBR nucleic acid florescence were observed within the EhV-86 population of particles.

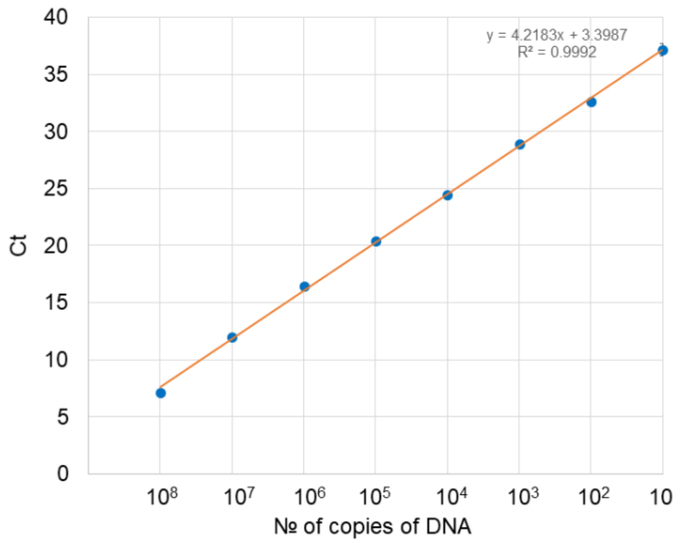

Fig S2. Example of a standard curve used in EhV qPCR assays. Dilution of the MCP PCR amplicon (no of copies) plotted against the Ct values as obtained by real time PCR (each dot represents three technical replicates). The Ct (cycle threshold) is defined as the number of cycles required for the fluorescent signal to cross the threshold (i.e., exceeds background level). The Ct was 7.16 for our 10^8 copies of target MCP stock, and it gradually increased to a final value of 37.16 in the presence of only 10 MCP copies. The R^2 value is shown.

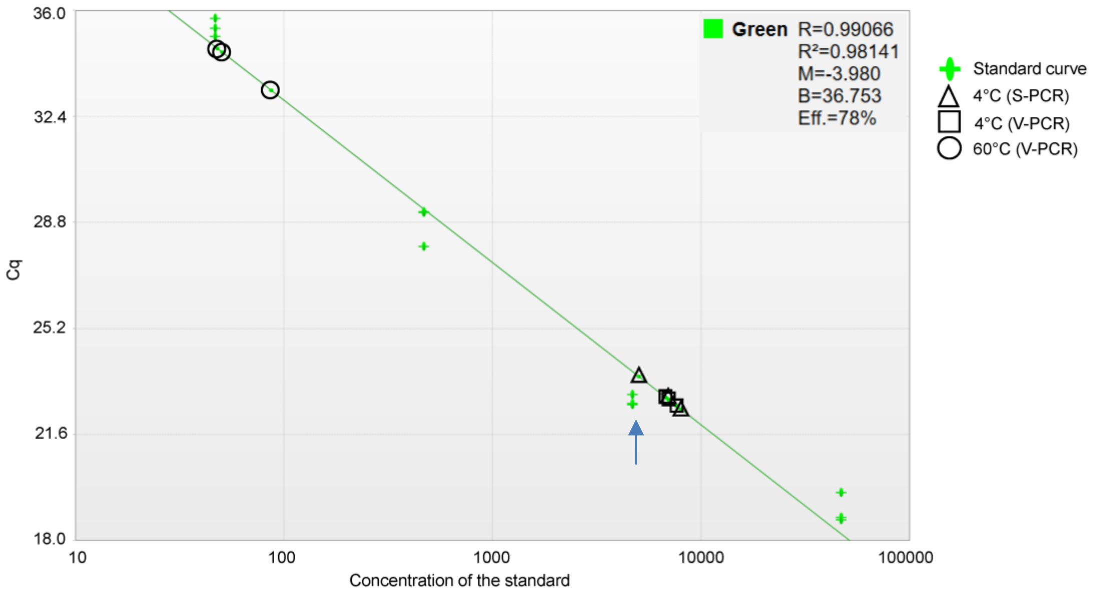

Fig S3. The standard curve (green line) created using a PRRSV $TCID_{50}$ mL^{-1} [3] 10-fold dilution series (green plus sign symbols, x-axis) and the Cq values (y-axis) obtained from RT-PCR. The Cq is defined as the number of cycles required for the fluorescent signal to cross the threshold (i.e., exceeds background level). The standard curve equation metrics are shown. The data obtained for Figure 3D are also shown. Note that the efficiency of the RT-PCR was only at 78% and that the 4 °C controls (triangles and squares) plotted on the standard curve gave higher $TCID_{50}$ mL^{-1} values than the corresponding standard dilution (arrow). It is clear from the plot that the RT-PCR is inhibited at the higher PRRSV concentrations. Consequently, our calculated $TCID_{50}$ mL^{-1} values for the S and V RT-PCR will be higher (just under 1 log) than the known starting $TCID_{50}$ mL^{-1} value (Figure 3D).

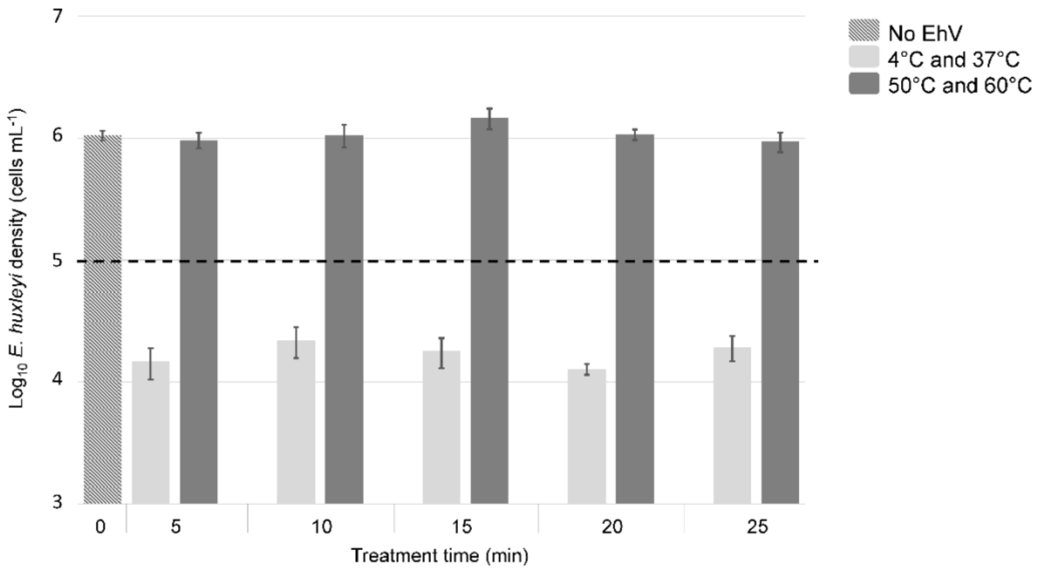

Fig S4 Infectivity of EhV-86 after temperature treatment. A $\log_{10}$ *E. huxleyi* cell density (cells mL^{-1}) plot indicating the capacity of EhV to induce *E. huxleyi* lysis, with subsequent change in cell density in infected cultures eight days post-virus inoculation in bioassays. EhV-86 was treated for short time-intervals: 5 min up to 25 min at several temperatures from 4 °C to 60 °C. The dashed line represents the lowest accurate limit of detection for cell number enumeration.

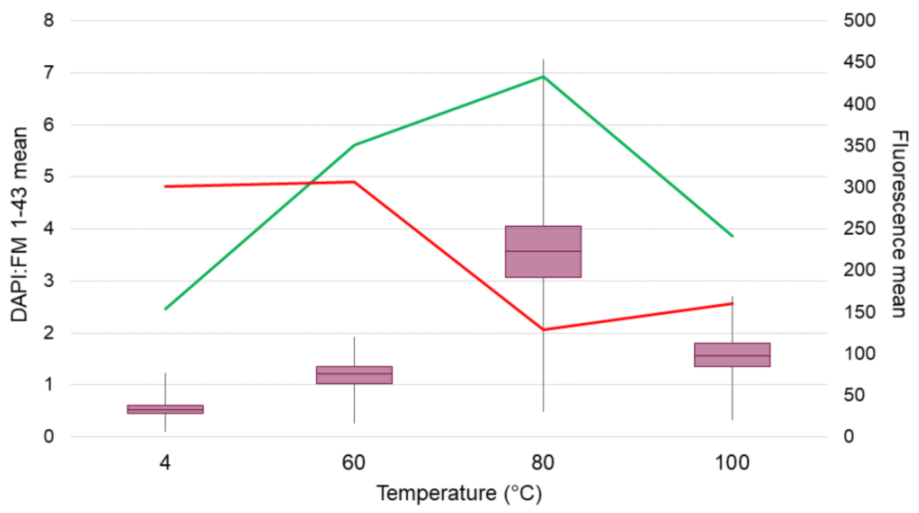

Fig S5. Efficiency of staining EhV-86 at different temperatures using a nucleic acid (DAPI) and lipid (FM 143) stain. Box plot with minimum & maximum (vertical whiskers), sample median (horizontal line) and first (25th percentile) and last (75th percentile) calculated for the nucleic acid and lipid membrane ratios (mean DAPI:FM 143 ratio) for EhV-86 treated for 20 minutes at 4 °C, 60 °C, 80 °C and 100 °C. Line plots of the individual DAPI (green line) and FM 143 (red line) mean fluorescence measurements for these aforementioned treatments are shown**.**

References

1. Marie D, Brussaard CPD, Thyrhaug R, Bratbak G, Vaulot D: **Enumeration of marine viruses in culture and natural samples by flow cytometry.** *Appl Environ Microbiol* 1999, **65:**45-52.
2. Martínez JM, Schroeder DC, Larsen A, Bratbak G, Wilson WH: **Molecular dynamics of *Emiliania huxleyi* and cooccurring viruses during two separate mesocosm studies.** *Applied and Environmental Microbiology* 2007, **73:**554-562.
3. Lei C, Yang J, Hu J, Sun X: **On the Calculation of TCID(50) for Quantitation of Virus Infectivity.** *Virologica Sinica* 2021, **36:**141-144.
